# LEV-10 and NRA-2 regulate distinct mechanisms of cholinergic tachyphylaxis in Caenorhabditis elegans

**DOI:** 10.21203/rs.3.rs-11130925/v1

**Published:** 2026-10-27

**Authors:** Shruti Gupta, Jacob Wragge, Sudhanva Kashyap

**Affiliations:** Creighton University; Creighton University; Creighton University

**Keywords:** Levamisole, anthelmintics, C. elegans, drug resistance, L-AChRs, cholinergic tachyphylaxis

## Abstract

Soil-transmitted helminthiasis affects more than a billion people worldwide and is a global health concern. With no effective vaccination available, treatment relies on mass administration of anthelmintic drugs, like albendazole, ivermectin, and levamisole. Unfortunately, their regular of overuse has given rise to resistance. Levamisole, a cholinergic agonist, activates the nematode L-type acetylcholine receptors and causes muscle contraction and worm paralysis. In parasitic nematodes, such as female *Brugia malayi*, worms paralyze and recover through cholinergic tachyphylaxis. In this study, we dissect the molecular mechanisms behind cholinergic tachyphylaxis using the free-living model, *C. elegans*. Here, we differentiate between two independent recovery mechanisms: one that is mediated by L-AChR clustering mutants – *lev-10* and another, mediated by an ER-resident protein, NRA-2. Cholinergic tachyphylaxis observed in *nra-2* mutants can be distinguished from *lev-10* mutants using motility assays, transcript analysis, and calcium imaging. *nra-2* mutants are more sensitive to levamisole and recover differently than *lev-10* mutants. We observe that *nra-2* mutants do not show increased calcium influx during their levamisole-mediated recovery. Our study delineates two distinct modes by which worms can adapt and overcome effects of cholinergic anthelmintics.

## Introduction

Soil-transmitted helminthiasis (STH), a group of neglected tropical diseases (NTDs), are a major health concern in tropical countries of the world, especially in countries with poor sanitation. In 2021, approximately 600 million STH cases were reported worldwide ^1^, mainly impacting young children ^2,3^. STH affects both humans and livestock, impacting the lives of infected individuals and the food industry, respectively. Current treatment for STH includes mass drug administration of benzimidazoles such as albendazole, macrocyclic lactones like ivermectin, and nicotinic agonists such as levamisole ^4,5^. These anthelmintics are ineffective in killing the adult worms ^6–8^, and thus multiple doses may be required to control the infection, increasing the risk of anthelmintic resistance ^9^. There is an urgent need to understand the mechanisms of resistance as well as to develop anthelmintics that effectively clear the adult parasitic nematode burden from the host.

Levamisole, an old and classical anthelmintic identified in the early 1960s ^10^, is primarily used against gastrointestinal nematodes, targeting the L-type acetylcholine receptors (L-AChRs). These receptors are heteromeric pentamers consisting of individual subunits. The parasitic L-AChR consists of ACR-8, UNC-38, UNC-29, and UNC-63 ^11^. Studies using the parasitic nematode *Brugia malayi* show that lower concentrations of levamisole produce efficient paralysis, but at higher concentrations, the worms regain motility despite the presence of the anthelmintic due to cholinergic tachyphylaxis ^12^. Levamisole activates L-type receptors, resulting in an increase in cytosolic calcium, inducing muscle contraction and worm paralysis^13^.Kashyap et al, 2021 reported downregulation of *nra-2* transcripts in the presence of levamisole, which then gradually returned to baseline upon removal of levamisole. These findings implicate the transient downregulation of *nra-2* in driving worm recovery in the presence of levamisole. NRA-2 is an endoplasmic reticulum (ER) protein that shares homology with the human protein, Nicalin ^14^. NRA-2 is involved in AChR subunit selection and their expression in muscle. NRA-2 recognizes and selects the proteins that can exit the ER based upon their tertiary or quaternary structure. NRA-2 controls the muscle nAChR composition, which affects the pharmacology of the parasitic muscle ^15^.

The free-living nematode *C. elegans* is widely used for studying anthelmintic resistance ^16^. *C. elegans* belongs to clade V nematodes and is closely related to major gastrointestinal nematodes. These nematodes have two types of L-AChRs, which are both found in the body wall muscle and neurons; while they share four of their subunits, they differ in their expression of one key subunit. The four shared subunits are: UNC-63, UNC-38, UNC-29, and LEV-1 ^17^. The fifth subunit is what distinguishes them: the canonical L-type receptor consists of LEV-8, while ACR-8 reconstitutes a receptor that is less sensitive to levamisole ^18,19^.

Clustering of receptors is critical for efficient synaptic transmission ^20^. L-AChR clustering is mediated by genes *lev-9*, *lev-10*, and *oig-4*. Synapse localization and anchoring of L-AChRs at the neuromuscular junction (NMJ) is mediated through OIG-4 ^21^. LEV-10 is a transmembrane protein ^22^ that, along with LEV-9, an extracellular scaffolding protein ^23^, anchors L-AChRs to the NMJ ^24^. In *lev-9* mutants, basal expression of *lev-10* was observed ^23^. However, *lev-9* expression was completely abolished, highlighting the key role of LEV-10 in recruiting LEV-9 and regulating the clustering of L-AChRs at the NMJ.

Levamisole produces maintained spastic paralysis of wild-type (WT) worms, whereas the L-AChR clustering mutants, such as *lev-9* and *lev-10*, paralyze and recover even at high concentrations of levamisole ^25,26^. *lev-10* mutants are insensitive to lower concentrations of levamisole ^22^. The disruption of L-AChR clustering in *lev-10* mutants reduces levamisole sensitivity and increases tachyphylaxis (adaptation) in *C. elegans*.

Calcium plays an important role in the *lev-10* tachyphylaxis — the cytosolic calcium increases for several hours during levamisole exposure, leading to loss of motility in worms ^26^. However, in L-AChR clustering mutants, calcium levels remain high for over two hours ^25,26^, resulting in activation of calcineurin and reduction in L-AChRs at the muscle cell membrane. This enables the worms to recover in high concentrations of levamisole ^25,26^. In *B. malayi*, recovery is attributed to reduced expression of *nra-2* ^12^, but the effect of levamisole on the clustering machinery is unknown. Importantly, the correlation between the parasitic worm recovery through ER retention and the free-living worm recovery through inhibition of L-AChR clustering is yet to be deciphered

In this study, we differentiated two independent modes of worm recovery in *C. elegans* mediated by: (i) inhibition of NRA-2 and ER retention; and (ii) the disruption of the LEV-9/10 L-AChR clustering complex. In our study, we observed dose-dependent paralysis and robust recovery in *nra-2* null mutants in the presence of levamisole. Additionally, *nra-2* transcript levels remained unchanged in *lev-9* and *lev-10* mutants. Further, levamisole treatment resulted in reduced intracellular calcium release in *nra-2* mutants compared to *lev-10*. Our study suggests that ER retention and altered membrane trafficking of AChRs could be the key drivers of cholinergic tachyphylaxis in *nra-2* mutants.

## Results

### nra-2 mutants exhibit dose-dependent paralysis followed by recovery in the presence of levamisole.

Here, we use *C. elegans* as a model to study cholinergic tachyphylaxis and differentiate among molecular mechanisms behind worm recovery between mutants. Levamisole causes muscle paralysis; hence, we initially looked at the effects of levamisole on different mutants. All the worms – N2 (wildtype), *lev-10 (kr26::Mos1)*, *lev-9 (ok2166)*, and *nra-2 (ok1731)* were exposed to different concentrations of levamisole (1mM to 30μM) for 18 hours, and the motility was tracked using wMicrotracker^™^. *lev-10* mutants were paralyzed, followed by robust recovery at 1mM levamisole at 240 minutes (Fig. 1C). At lower levamisole concentrations, *lev-10* mutants displayed no sensitivity and were resistant to levamisole as described previously ^25^. *lev-9* mutants exhibited modest recovery in the presence of levamisole (Fig. 1B), unlike the *lev-10* mutants. *nra-2* mutants display initial sensitivity similar to wild-type; it is observed that *nra-2* mutants recover after 18 hours, contrary to wild-type (Fig. 1D).

### Levamisole has concentration-dependent inhibitory effects on C. elegans mutants.

Muscle contraction and worm paralysis were observed in both filarial nematodes and *C. elegans* treated with levamisole. In *Brugia malayi*, levamisole caused robust and dose-dependent worm paralysis with a very low IC_50_ of 10 ± 2.2 nM ^12^. We sought to characterize the inhibitory effects of levamisole across different *C. elegans* mutants to determine whether differences in the initial drug response could distinguish their subsequent recovery phenotypes. An IC_50_ plot at maximum decrease in motility, 150 minutes, was generated. The IC_50_ plot represents the concentration response curves for the paralytic effect of levamisole plotted against the percent motility of worms in the presence of levamisole (Fig. 2A). For IC_50_ curves, drug concentrations were transformed into log_10_ scale before analysis. The log inhibitor vs. motility responses were used to generate concentration response curves to calculate IC50 values. The IC_50_ of N2 and nra-2 mutants were similar, 0.079 ± 0.022mM and 0.077 ± 0.011mM, respectively. The IC_50_ for *lev-10* and *lev-9* mutants was significantly higher, 0.52 ± 0.07 mM and 0.18 ± 0.03mM respectively, and were partially resistant to levamisole.

### Distinct paralysis and recovery kinetics among C. elegans mutants.

Paralytic effects of levamisole on wild-type and *nra-2* mutants are similar, but we observe that *nra-2* mutants recover by the end of 18 hours in the presence of levamisole when compared to wild-type. Therefore, we assessed the kinetics of paralysis and recovery profiles of wild-type and other mutants in the presence of levamisole. We calculated the coefficient of paralysis, K_p_ in 1mM levamisole for *lev-10* = 0.01301 min^− 1,^ whereas N2 = 0.03185 min^− 1^ and *nra-2* = 0.04606 min^− 1^ (Fig. 3A) and at 300μM levamisole K_p_ for *lev-10* = 0.0009269 min^− 1^, N2 = .02813 min^− 1^ and *nra-2* = 0.01672 min^− 1^ (Fig. 3B). *lev-10* mutants have the lowest K_p_ values, indicating that these mutants undergo the least paralysis at both concentrations when compared to other strains. The K_p_ values are representative of the individual strain motility profile shown in Fig. 1.

Recovery slope at 1mM lev for N2 = 0.00067901 min^− 1^, *lev-10* = 0.163975 min^− 1^ and *nra-2* = 0.0094972 min^− 1^ (Fig. 3C) and at 300μM lev for N2 = 0.019363 min^− 1^, *lev-10* = 0.051515 min^− 1^ and *nra-2* = 0.07854833 min^− 1^ (Fig. 3D). *lev-10* has the highest slope values at both observed concentrations of levamisole, indicating that *lev-10* mutants recover robustly in the presence of levamisole, as depicted in the motility assays. Using these kinetic studies, we observe that despite *nra-2* mutants exhibiting an IC_50_ similar to that of wild-type, their recovery profile is different. Furthermore, *nra-2* mutants display paralysis and recovery profiles different from *lev-10* mutants, indicating that these mutants exhibit distinct responses to levamisole exposure.

### lev-9/10 mediated recovery is not mediated by a change in nra-2 expression.

In *B. malayi*, worm recovery in the presence of levamisole was attributed to a change in L-AChR composition at the muscle membrane, driven by downregulation of nra-2. But the role of l*ev-9/10* was unknown in *B. malayi* motility recovery. We describe worm recovery in *nra-2* and *lev-9/10* mutants in the presence of levamisole, due to cholinergic tachyphylaxis (Fig. 1). Quantifying the *nra-2* levels could elucidate the role of *nra-2* in *lev-9/10* mediated recovery. First, transcript levels were analyzed without levamisole, where no significant change in *nra-2* transcript in *lev-9 and lev-10* mutants (Fig. 4A) and *lev-9 and lev-10* transcripts in *nra-2* mutants was observed (Fig. 4B). We continued the transcript analysis in the presence of 1mM levamisole for 18 hours. No significant changes in either *nra-2* or *lev-9 and lev-10* genes were detected (Fig. 4C, S1 and 4D). These results highlight that transcriptionally, *nra-2* and *lev-10* do not interact with each other in their respective worm recovery mechanism.

### Worm recovery in nra-2 mutants is independent of calcium homeostasis mechanisms.

In a previous study, it was reported that the recovery in *lev-10* mutants was accompanied by an increase in intracellular calcium levels, which persists up to four hours ^25^. We wanted to ask the question: are elevated calcium levels necessary for recovery in *nra-2* mutants? Therefore, we monitored the change in cytosolic calcium in the body wall muscle for 18 hours in the presence of levamisole.

We used a transgenic strain expressing a calcium indicator tagged with GFP, GCaMP3(*goels3*), in the body wall muscle. We generated double mutants by crossing *nra-2* and *lev-10* mutants with GCaMP3. Worms were exposed to 1mM levamisole for 18 hours, and calcium levels in the body wall muscles were measured at 0th, 2nd, 4th, and 18th hours. Initially, in the absence of levamisole, *lev-10* had reduced calcium levels compared to wild type, whereas the calcium level in the body wall muscle of *nra-2* mutants was similar to the control *goels3* worms (Fig S2). When the worms were exposed to levamisole, we observed a sudden increase in GFP in *lev-10* mutants, whereas the fluorescence intensity in *nra-2* mutants was similar to wild-type (Fig. 5A). Over a period of 18 hours, we observed that *lev-10* continued to have higher calcium levels than *nra-2* and wild-type. Interestingly, we observed that *nra-2* worms recover despite having a calcium profile similar to that of the wildtype (Fig. 5B). Altogether, these results suggest that *nra-2* has a recovery mechanism completely different from the *lev-10* recovery mechanism in *C. elegans*.

## Discussion

Levamisole resistance and worm recovery have been described in several studies ^27,28^, but the molecular mechanisms underlying this resistance are still unknown. *C. elegans* are widely used to study the impact of levamisole on parasitic nematodes. Here, we compare the widely known recovery mechanism via L-AChR clustering genes and another recovery mechanism mediated by *nra-2*. Clustering of receptors is essential for generating stronger signals, leading to better cellular responses ^29^. Parasitic nematodes consist of various families of receptors, such as glutamate-gated receptors (GluCls), transient receptor protein channels (TRP), and nicotinic acetylcholine receptors (nAChRs) ^16^. In *C. elegans*, nAChRs are made up of UNC-38, UNC-63, UNC-29, LEV-1, and LEV-8 ^16^. *lev-10* is a transmembrane protein that localizes to the NMJ ^26^ and is required for proper L-AChR clustering ^21^. Mutations in *lev-10* result in defective L-AChR clustering and a reduction in levamisole sensitivity.

Nuclear hormone receptors (NHR) are transcription factors that regulate biological activities ^30^. They can modulate the expression of various genes involved in anthelmintic resistance ^30^. Ivermectin resistance is associated with upregulation of P-glycoproteins (PGPs) in parasitic nematodes ^31^. NHR-8 has been linked to upregulation of PGPs contributing to ivermectin resistance in parasites using *C. elegans* as a model ^32^. The ability of parasitic nematodes to alter gene expression based on the presence of a drug does not involve a single gene; multiple genes are part of the same mechanism. Similarly, downregulation of *nra-2* observed in *B. malayi* in the presence of levamisole, followed by *nra-2* levels returning to the basal levels after removal of levamisole ^12^, could be due to modulation of *nra-2* by a nuclear hormone receptor.

NRA-2, a protein in the ER, helps distinguish channel conformations and prevents improperly assembled receptors from exiting the ER ^14^. It functions as a gatekeeper for receptor subunits that can be expressed as part of L-AChRs on the cell membrane. We observed that *nra-2* mutants exhibit cholinergic tachyphylaxis when exposed to levamisole. The differing recovery patterns between *nra-2* and *lev-10* mutants may stem from their specific cellular roles. Additionally, *nra-2* shares homology with human nicalin, but uniquely contains an EF hand motif ^14^. EF hands are helix-loop-helix structures ^33^ found in many ER proteins that use calcium binding to regulate protein sorting and folding ^34^. One of the roles of the EF-hand motif is calcium ion binding ^35^. For example, STIM1, crucial for store-operated calcium entry (SOCE) ^36^, uses its EF-hand motif to detect ER calcium levels ^37^ and activates calcium release-activated calcium channels (I_CRAC_) ^38^. Mutations in the EF-hand motif can impair calcium sensing by STIM1, causing continuous SOCE even when ER calcium is high ^39^. Changes in EF-hand structure thus influence protein function. We speculate that the *nra-2* mutation disrupts EF-hand function, leading to the translocation of levamisole-insensitive receptors to the muscle membrane, resulting in cholinergic tachyphylaxis. Loss of *nra-2* has been linked to increased ACR-8-containing receptors on muscle membranes ^15^. This increase could result from impaired EF hand activity caused by reduced ER calcium, with the EF hand potentially acting as a ‘dock’ for ACR-8 receptors that fails at reduced calcium levels.

One notable difference between the intestine and cuticle is the expression and distribution of P-glycoproteins (PGPs). ^40^ reported increased expression of *pgp-11* in the intestine of both male and female *Parascaris equorum* compared with body-wall muscle. Similarly, in *C. elegans*, expression of *pgp-9* in the intestine and cuticle has been implicated in modulating susceptibility to the macrocyclic lactones ivermectin and moxidectin ^41^. PGPs belong to a superfamily of ATP-binding cassette (ABC) transporters and they utilize ATP hydrolysis to translocate various substrates across membranes ^42^. It is speculated that ABC transporters could be involved in causing the efflux of anthelmintics away from their target site in parasitic nematodes and promoting worm recovery ^43^. Consistent with this hypothesis, PGPs have been implicated in ivermectin resistance ^32^ and intestinal expression of PGPs has been described in both *C. elegans* ^44^ and parasitic nematodes such as *H. contortus* ^45^. Collectively, these findings raise the possibility of PGPs playing a potential role in levamisole efflux from the intestine, enabling worm recovery.

Differences in L-AChR subunits between parasitic nematodes and *C. elegans* may cause variations in IC50 values. A notable distinction is that parasitic nematodes include the ACR-8 subunit in their L-AChR composition, whereas *C. elegans* lacks this subunit. ACR-8 is involved in levamisole sensitivity ^46,47^ and calcium sensitivity in parasitic nematodes ^47^. Recently, a SNP in ACR-8, S168T, was reported in a levamisole-resistant parasitic nematode, *Haemonchus contortus* ^48^. Interestingly, ACR-8 in *C. elegans* consists of threonine at the 168th position instead of serine. A recent study reported that levels of ACR-8 in the muscle membrane were a lot higher than previously described ^19^. This implicates a more regulatory role for ACR-8-containing L-AChRs in C. elegans. The presence of ACR-8 S168T instead of wild-type ACR-8 (S168) leads to changes in levamisole sensitivity in parasitic nematodes ^18^. It could be hypothesized that increased presence of ACR-8 (168T) in *C. elegans* may contribute to increased IC50 and decreased sensitivity compared to the parasitic nematodes.

Levamisole activation of L-AChR leads to the entry of calcium and causes an initial rise in calcium. Upon activation of acetylcholine receptors, the membrane potential depolarizes, triggering the opening of voltage-gated calcium channels (VGCCs) and ryanodine receptors (RyR) in the muscle ER, permitting calcium influx ^49^. Increased intracellular calcium induces CICR ^50^. *lev-10* mutants adapt to levamisole exposure due to an increase in cytosolic calcium, followed by CICR and emptying of the calcium stores in the ER due to homeostatic mechanisms ^12,26^. It can be speculated that *lev-10* mutants follow the ryanodine-sensitive pathway ^49^ to increase calcium influx, followed by calcium homeostasis, which promotes locomotion recovery. In *nra-2* mutants, we do not observe a sustained calcium release during worm paralysis and recovery, indicating that *nra-2* mutants do not empty their calcium stores during levamisole recovery.

Loss of *nra-2* leads to the translocation of L-AChRs consisting of receptors such as ACR-16, ACR-8, and UNC-29 to the cell membrane ^15^. ACR-8 has been identified in purified L-AChR along with seven other subunits – UNC-38, UNC-29, UNC-63, LEV-1, LEV-8, and ACR-12. ACR-8 shares homology with LEV-8 ^51^ and both can compensate for each other ^19^. We speculate that *nra-2*-mediated recovery is driven by translocation of ACR-8-containing receptors to the membrane, which renders the worms insensitive to levamisole. Recently, it has been found that ACR-12 can replace ACR-8 and LEV-8 to form complexes with UNC-29 ^19^. ACR-12 belongs to the same subfamily as ACR-8 and LEV-8 ^52^. It is an α-subunit of ionotropic acetylcholine receptors and is involved in the synaptic drive and sinusoidal motor pattern ^53^. These studies demonstrate diversity in receptor composition in *C. elegans* similar to parasitic nematodes, and depending upon the changes in their pharmacological environment, they can potentially alter their membrane pharmacology to survive the paralytic effects of cholinergic anthelmintics.

## Conclusion

Soil-transmitted helminthiasis affects the lives of affected individuals and livestock production. The inefficiency of current anthelmintics towards adult parasitic worms promotes worm recovery and adaptation, especially in the presence of cholinergic anthelmintics. Here, we highlight two possible mechanisms that could contribute to worm recovery in the presence of levamisole, mediated through LEV-10 and NRA-2, and differentiate the molecular mechanisms between them. We report that these two mechanisms function independently of each other to aid worm recovery in the presence of levamisole. Studying the recovery mechanism is essential and needed in real time because it will promote the development of anthelmintics that target those mechanisms and paralyze the worm efficiently. With the rising anthelmintic resistance, identifying and targeting recovery mechanisms represents a promising approach for next-generation drug development. Future studies on these mechanisms will accelerate the development of effective therapeutic interventions.

## Data Availability

The datasets generated during the current study are available from the corresponding author upon reasonable request.

## Methods

### Nematode maintenance

*C. elegans* used in this study were obtained from the Caenorhabditis Genetics Center (CGC, University of Minnesota, Minneapolis, USA). All worms were grown on *E. coli* OP50 at 20°C using established methods provided in the WormBook ^54^. Genetic crosses were performed to generate double mutants.

### Motility assays

One day old young *C. elegans* adults were used for motility assays. Worms were washed thrice with M9 buffer supplemented with 0.1% bovine serum albumin (BSA). Approximately 150–250 worms were used for these assays. WMicrotracker Arena (PhylumTech, Santa Fe, Argentina) was used to measure the motility for 18 hours at 20°C. WMicrotracker principle is explained in Risi et al., 2019, and Simonetta and Golombek, 2007 ^55,56^. Briefly, the system is optimized to quantify the motility of small organisms in a six- or twenty-four well plate. It is equipped with a Peltier system that controls the temperature. The system detects the worm motion through the infrared light scattering effect. The detection area is composed of multiple infrared microbeams that illuminate from the bottom, while a six-camera video optical system captures the light that passes through the worms. The analogue output is recorded and the signal from each microbeam is acquired and processed mathematically to detect the light scattering by worm motion. When motion is detected, the activity counter is incremented.

### Paralysis and recovery kinetics

We used customized nonlinear regression equations in GraphPad Prism to fit the paralysis and recovery kinetics. For the paralysis rate (Kp), we used an explicit equation -

$$Y=\mathrm{Plateau}+(100-\mathrm{Plateau})e^{\left(-Kp\;X\right)}$$

where Y - motility %, plateau - saturation of paralysis and X – time for paralysis (X<240mins).

We studied the recovery kinetics by identifying the slope of the curves using the slope-intercept form of a linear equation –

Y=slope×X+intercept

where Y - motility % and X – time for recovery (X > 180 mins for N2 and *nra-2* and X > 120 for *lev-10*).

### RNA extraction and qPCR

RNA extraction was performed using TRIzol protocol explained in ^12,57^. RNA from *C. elegans* was extracted by snap-freezing the worms in liquid nitrogen and heat shock. 1μg of RNA was used to synthesize cDNA using SuperScript^™^ VILO^™^ (Thermofisher Scientific, USA). cDNA from both treated and untreated samples was amplified using target and reference (*gapdh*) primers. List of primers used in this study are provided in supplementary table 1.

Gene amplification was performed in triplicate using QuantStudio. Relative quantification of target cells was estimated using ΔΔ Ct method.

### Calcium Imaging

Age-synchronized worms were exposed to levamisole for 18 hour. Imaging was performed at intervals of 2 hours and finally at 18 hours. We picked 4–5 worms at every time point and placed them in 2% agarose pads consisting of the same concentration of levamisole they were exposed to before. We immobilized the worms using a coverslip and M9 buffer. Imaging was conducted using the Fiji plugin provided by Nikon Elements AR to quantify the change in calcium levels. We performed the Fourier transformation using the software embedded in Nikon AR Elements to remove the background noise. Region of interest (ROI) was detected along the edge of the worm, and the maximum intensity was noted. The maximum intensity of treated worms was normalized to the control worms at the given time point. The change in calcium was plotted using GraphPad Prism.

### Statistical analysis

The statistical analysis was performed as explained in ^12,58^. The datasets were analyzed using GraphPad Prism 11.1.0 (Graphpad Software, Inc., La Jolla, CA, USA). For motility analysis, % motility was plotted against time (in minutes). For *the* levamisole concentration and inhibition effect relation on different mutants, drug concentrations were log_10_ transformed before analysis. The IC50 values and concentration responses were fitted using the log inhibitor vs. response equation (variable slope). Values were plotted as the mean±SEM. Two way ANOVA and Bonferroni post hoc test were done to test significance. As an alternative, Student T-test were used to determine whether the significance among the groups is appropriate. GraphPad Prism was used to perform statistical analysis.

## Supplementary Material

Supplementary Files

This is a list of supplementary files associated with this preprint. Click to download.

• Guptaetal2026Table1.xlsx

• Guptaetal2026FigS1.tiff

• Guptaetal2026FigS2.tiff

## Figures and Tables

**Figure 1:**
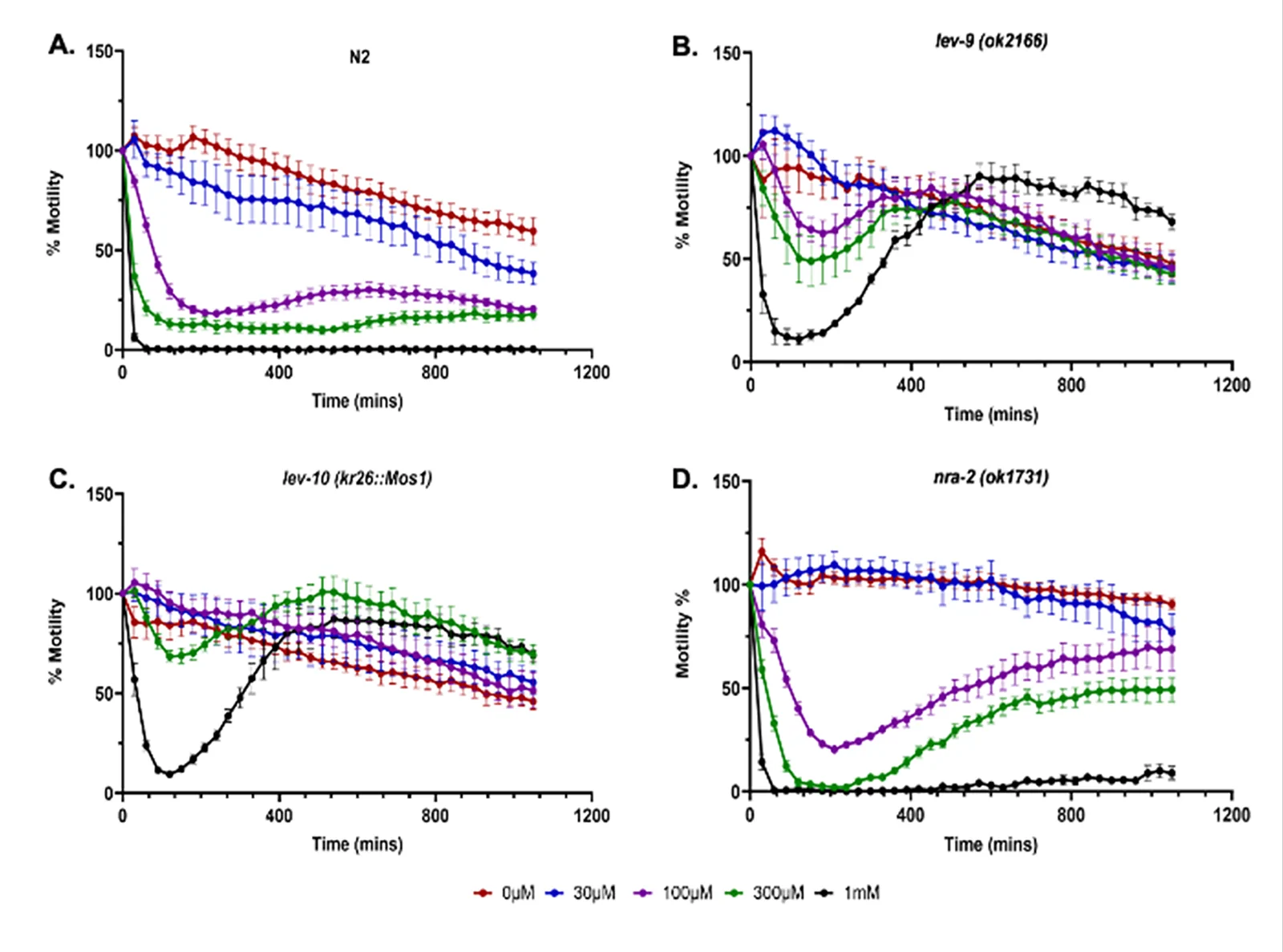
Levamisole recovery profile in *nra-2*mutants is distinct from *lev-9* and *lev-10* mutants. A) Dose-dependent paralysis of N2 in the presence of increasing concentrations of levamisole (red - 0μM, blue - 30 μM, purple - 100 μM, green - 300 μM, and black – 1mM). B) and C) Effect of increasing levamisole concentrations on *lev-9(ok2166)* and *lev-10(kr26::Mos1)*mutants, respectively. D) Dose-dependent paralysis and recovery of *nra-2(ok1731)*at increasing levamisole concentrations. At 1mM levamisole, p<0.0001 for *lev-9(ok2166)* and *lev-10(kr26::Mos1)* and p<0.005 for *nra-2(ok1731)*. At 300μM, p<0.0001 for *lev-9(ok2166)* and *lev-10(kr26::Mos1)* and p<0.005 for *nra-2(ok1731)*. At 100μM, p<0.0001 for *lev-9(ok2166),*p<0.005 for *lev-10(kr26::Mos1)* and *nra-2(ok1731);* two-way ANOVA. N=6 replicate studies for each strain and concentration, and n=150–250 worms/study.

**Figure 2:**
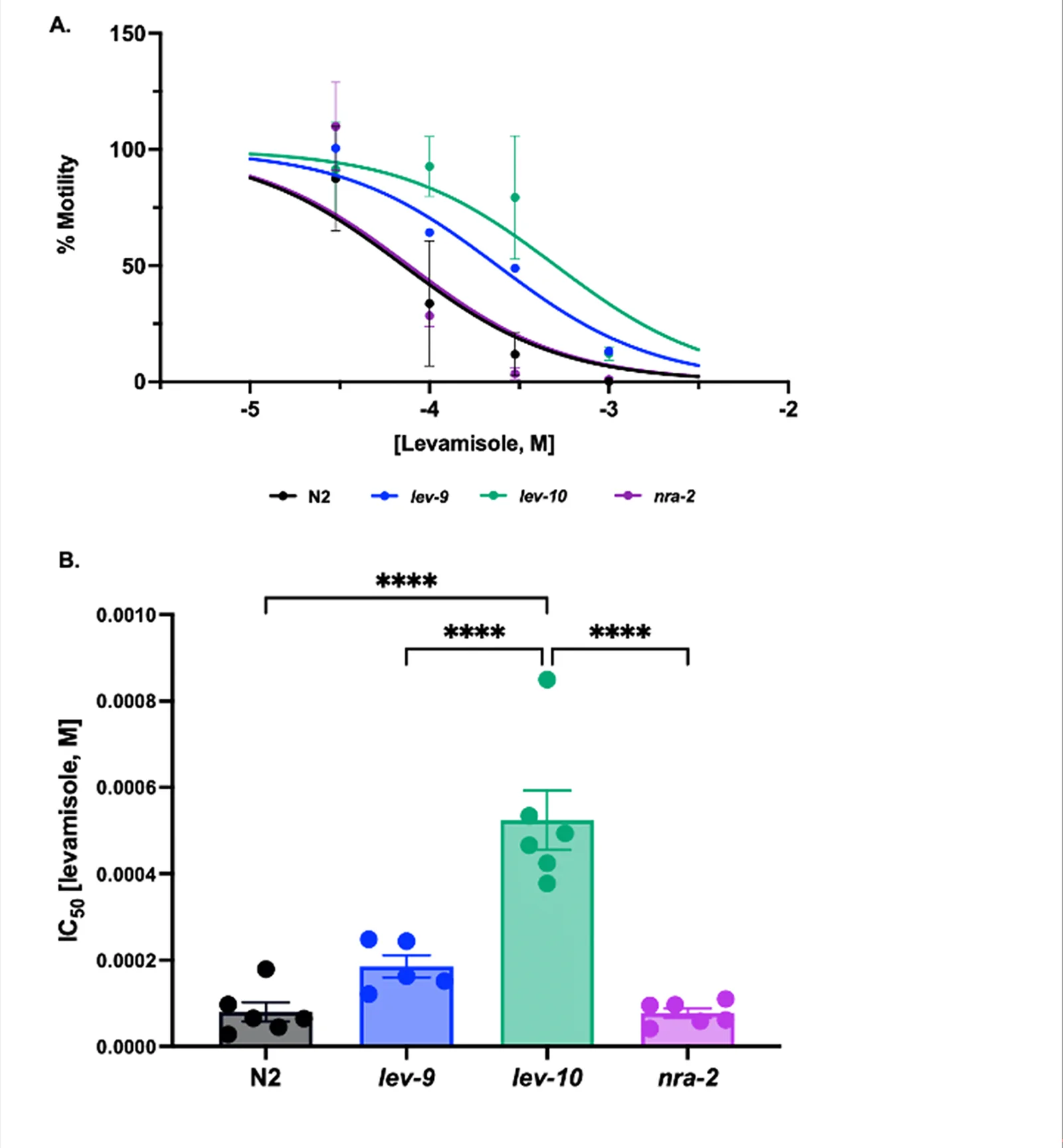
*nra-2* is more sensitive to levamisole than *lev-10*. Concentration-response curves for the paralytic effect of levamisole. IC_50_ measured at t150 for N2 – 0.079±0.022mM (black); *lev-9 (ok2166)* – 0.18±0.03mM (blue); *lev-10(kr26::Mos1)* - 0.52±0.07mM (green) and *nra-2 (ok1731)* – 0.077±0.011mM (purple). p<0.001 for N2 vs. *lev-10(kr26::Mos1)* and *lev-10(kr26::Mos1)* vs. *lev-9 (ok2166)*. **B)** Histogram representing the IC_50_ values measured at 150 minutes post-treatment with levamisole for the different strains. p<0.001 for N2 vs. *lev-10(kr26::Mos1)* and *lev-10(kr26::Mos1)* vs. *lev-9 (ok2166)*.

**Figure 3:**
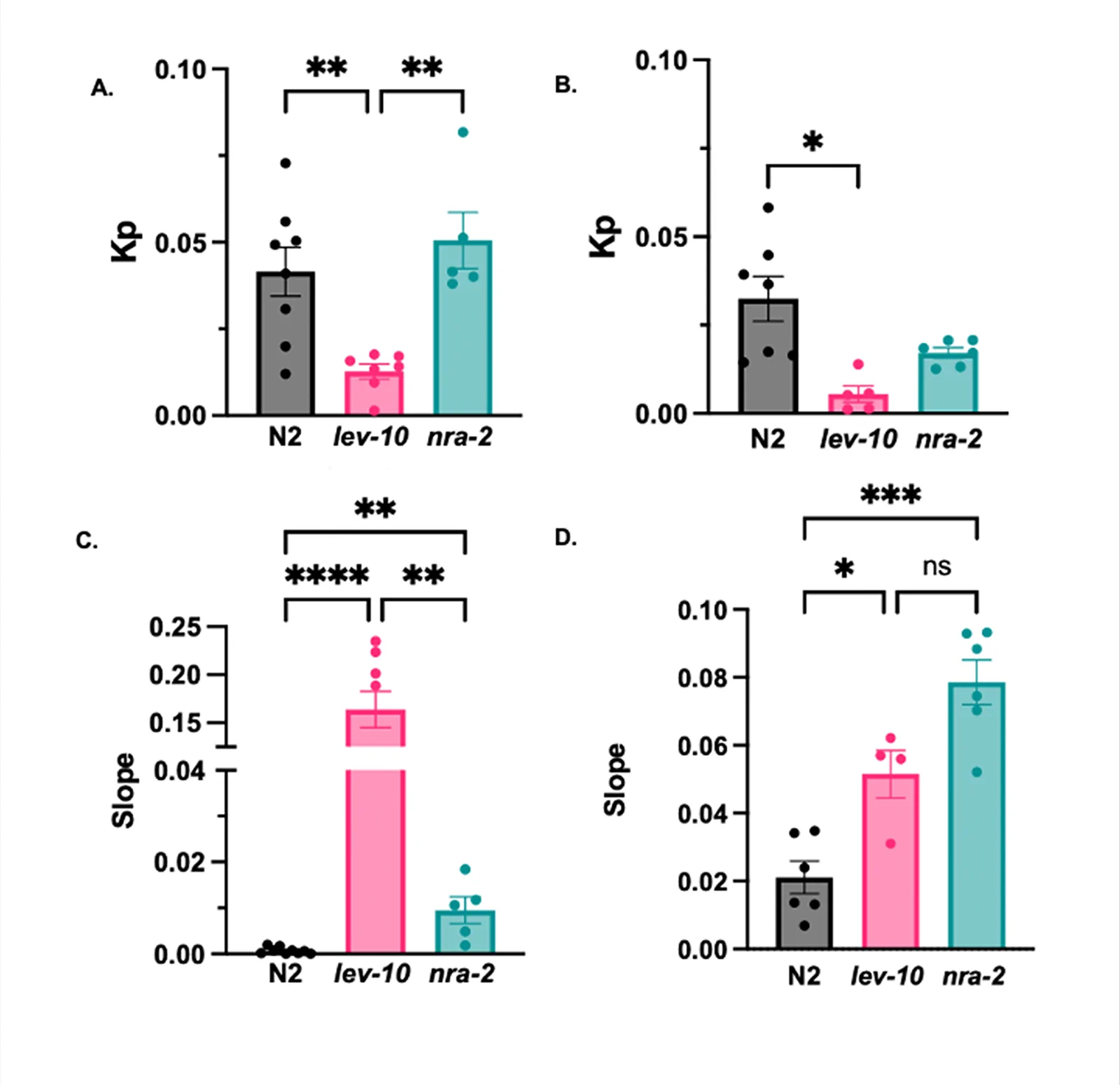
*nra-2* and *lev-10* mutants have distinct paralysis and recovery kinetics in the presence of levamisole. **A)** Differences in paralysis phenotype among the three mutants. The co-efficient of paralysis, K_p_ for 1mM lev, N2 = 0.03185 min^−1^, *lev-10* =0.01301 min^−1^, *nra-2* = 0.04606 min^−1^ **B)** K_p_ for 300μM lev – N2 = 0.02813 min^−1^, *lev-10* = 0.0009269 min^−1^ and *nra-2* = 0.01672 min^−1^. One–way ANOVA – p<0.005 for *lev-10* v/s N2 and *lev-10* v/s *nra-2* at 1mM and p<0.05 for *lev-10* v/s N2 at 300μM levamisole. **C)** Differences in recovery phenotypes between the mutants. Slope at 1mM levamisole for N2 = 0.00067901 min^−1^, *lev-10* = 0.163975 min^−1^ and *nra-2* = 0.0094972 min^−1^ **D)** Slope at 300μM levamisole for N2 = 0.019363 min^−1^, *lev-10* = 0.051515 min^−1^ and *nra-2* = 0.07854833 min^−1^. One-way ANOVA p<0.0001 for *lev-10* v/s N2, *lev-10* v/s *nra-2* and p<0.005 for *nra-2* v/s N2 at 1mM levamisole and p<0.0005 for *nra-2* v/s N2 and p<0.05 for *lev-10* v/s N2 at 300μM levamisole.

**Figure 4:**
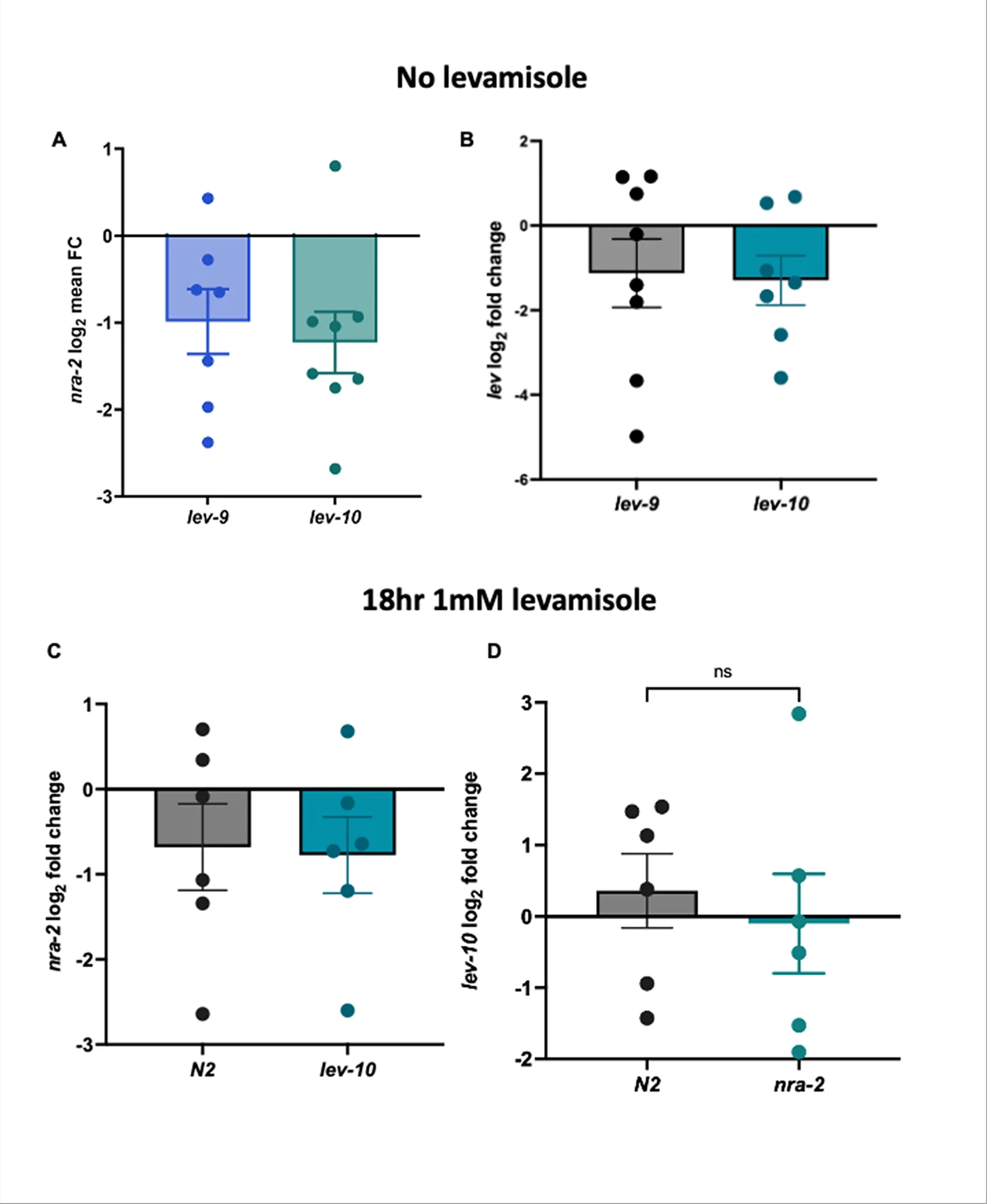
*nra-2* and *lev-10* mediate cholinergic tachyphylaxis through independent transcription pathways. **A)** Downregulation of *nra-2* transcripts in *lev-9* and *lev-10* mutants. log_2_ mean FC for *lev-9* = −0.874 and *lev-10* = −0.821, N=7. **B)** Change in *lev* transcript levels in N2 and *nra-2* mutants. log_2_ mean fold change for *lev-10* = −0.364 and *lev-9* = 0.171 and N=7. **C)** Change in *nra-2* transcript levels in N2 and *lev-10* mutants after 18 hours in 1mM levamisole. log_2_ mean fold change for N2 = 0.995 and *lev-10* = −0.467, N=6. p>0.05, Student’s t-test. **D)** Changes in *lev-10* transcript post-18-hour treatment with 1mM levamisole. log_2_ fold change for *nra-2* = 0.865 and N2 =0.427, N=6. Pre-levamisole amplification values were normalized to N2 (wild-type) *gapdh*. Post-levamisole treatment amplification values were normalized to the strain-specific *gapdh*.

**Figure 5:**
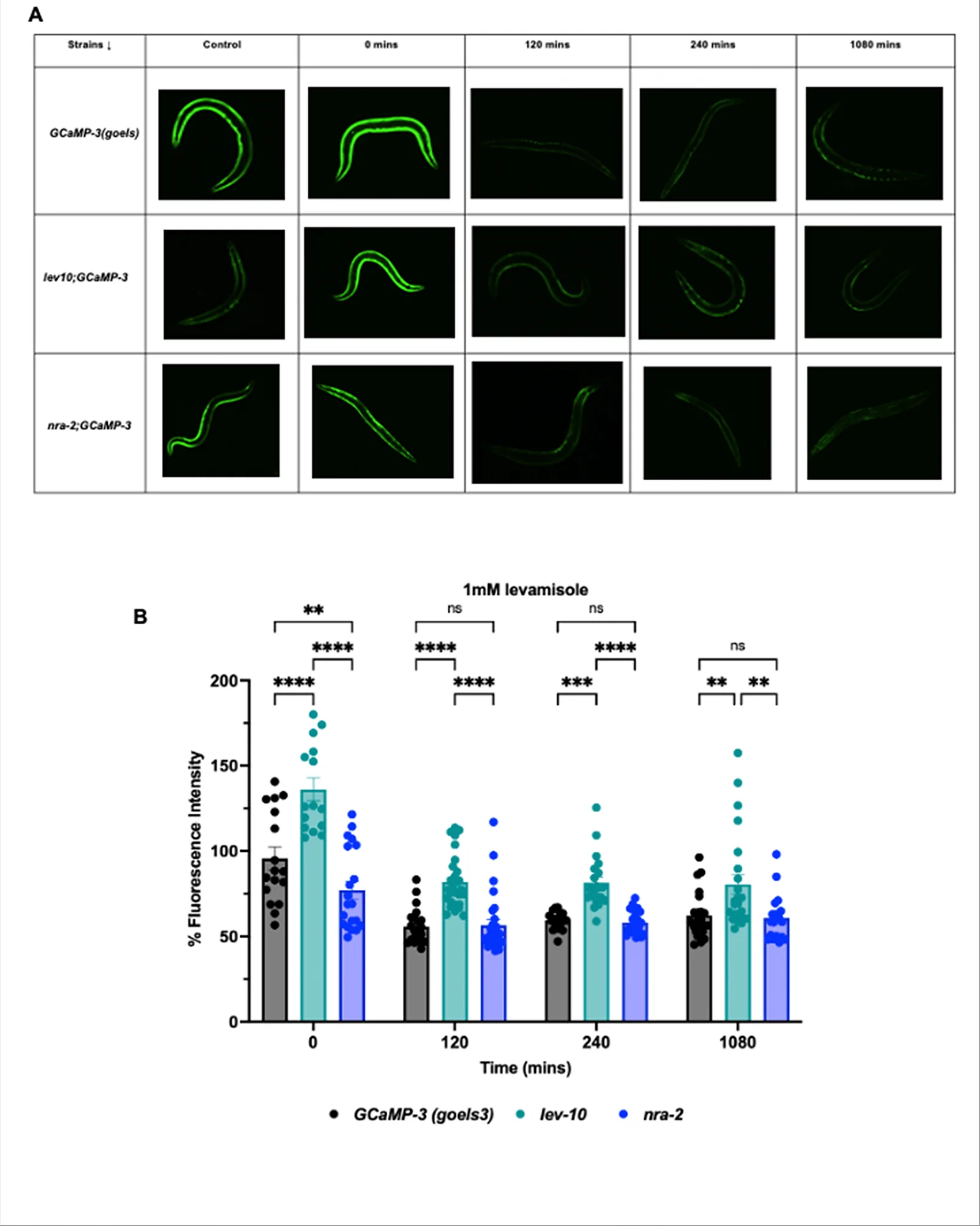
Levamisole-induced cholinergic tachyphylaxis is independent of calcium homeostasis in *nra-2* mutants. **A)**Images displaying influx of calcium in body wall muscle induced by 1mM levamisole in GCaMP3(*goels3*) (black), *lev-10;goels3* ( green) and *nra-2;goels3* (blue) animals after 0^th^ hour, 2^nd^ hour, 4^th^ hour, and 18^th^ hour. For control conditions, adult worms were imaged without levamisole. **B)** Histogram quantifying the cytosolic calcium levels in GCaMP-3(*goels3*), *lev-10,* and *nra-2* animals. At 0^th^ hour p<0.01 and NS at 2^nd^, 4^th^ and 18^th^ hour between *nra-2* and GCaMP-3. At 0^th^, 2^nd^, and 4^th^ hour p<0.0001 and p<0.01 for 18^th^ hour between *lev-10* and GCaMP-3. Two-way ANOVA and the percentage fluorescent intensity is plotted as mean± SEM by normalizing the mean intensity to the control. N=15–20 worms for each time point for each strain.

## Data Availability

The datasets generated during the current study are available from the corresponding author upon reasonable request.
